# Novel RNA aptamers targeting gastrointestinal cancer biomarkers CEA, CA50 and CA72-4 with superior affinity and specificity

**DOI:** 10.1371/journal.pone.0198980

**Published:** 2018-10-10

**Authors:** Qing Pan, Carmen O. K. Law, Mingo M. H. Yung, K. C. Han, Yuen Lam Pon, Terrence Chi Kong Lau

**Affiliations:** Department of Biomedical Sciences, City University of Hong Kong, Kowloon, Hong Kong Special Administrative Region, China; Consiglio Nazionale delle Ricerche, ITALY

## Abstract

Gastric cancer is the third most common cause of death from cancer in the world and it remains difficult to cure in Western countries, primarily because most patients present with advanced disease. Currently, CEA, CA50 and CA72-4 are commonly used as tumor markers for gastric cancer by immunoassays. However, the drawback and conundrum of immunoassay are the unceasing problem in standardization of quality of antibodies and time/effort for the intensive production. Therefore, there is an urgent need for the development of a standardized assay to detect gastric cancer at the early stage. Aptamers are DNA or RNA oligonucleotides with structural domain which recognize ligands such as proteins with superior affinity and specificity when compared to antibodies. In this study, SELEX (Systematic Evolution of Ligands by Exponential enrichment) technique was adopted to screen a random 30mer RNA library for aptamers targeting CEA, CA50 and CA72-4 respectively. Combined with high-throughput sequencing, we identified 6 aptamers which specifically target for these three biomarkers of gastrointestinal cancer. Intriguingly, the predicted secondary structures of RNA aptamers from each antigen showed significant structural similarity, suggesting the structural recognition between the aptamers and the antigens. Moreover, we determined the dissociation constants of all the aptamers to their corresponding antigens by fluorescence spectroscopy, which further demonstrated high affinities between the aptamers and the antigens. In addition, immunostaining of gastric adenocarcinoma cell line AGS using CEA Aptamer probe showed positive fluorescent signal which proves the potential of the aptamer as a detection tool for gastric cancer. Furthermore, substantially decreased cell viability and growth were observed when human colorectal cell line LS-174T was transfected with each individual aptamers. Taking together, these novel RNA aptamers targeting gastrointestinal cancer biomarker CEA, CA50 and CA72-4 will aid further development and standardization of clinical diagnostic method with better sensitivity and specificity, and potentially future therapeutics development of gastric cancer.

## Introduction

According to the statistics from World Health Organization 2015, Gastric cancer is the third most common cause of cancer-related death in the world [1]. Nevertheless, gastric cancer remains difficult to cure due to the lack of early detection biomarkers and most patients are diagnosed at late stage or with advanced disease. For the last decades, detection of biomarkers or tumor markers has been widely used in clinical management which assist the screening, diagnosis, prediction of prognosis and recurrence, and post-treatment monitoring. Common biomarkers for gastrointestinal cancer can be largely classified as carcinoembryonic antigen (CEA), and tumor associated antigens, such as cancer antigen 19–9 (CA19-9), cancer antigen 50 (CA50) and cancer antigen 72–4 (CA72-4) [2]. CEA, with a molecular weight of 180–200 kD, is a cell surface glycoprotein that plays a role in cell adhesion and intracellular signaling [3]. Cancer antigen 50 (CA50), with a molecular weight of ~210 kD, is defined by the monoclonal antibody C 50 developed against a colorectal cancer (CRC) cell line COLO-205. It has elevated levels in serum and can be observed in a variety of malignancies, especially gastrointestinal cancers [4]. Cancer antigen 72–4 (CA72-4), with a molecular weight of 220–400 kD, is a mucin-like high molecular weight tumor associated antigen, and it is considered as the first choice of tumor marker for gastric carcinoma because of a superior sensitivity than CEA and CA19-9 [1].

Currently, enzyme immunoassay (EIA) and radio-immunoassay (RIA) are commonly employed for the detection of tumor markers [2]. Although the interaction between antibodies and antigens are highly specific, the biggest drawback and conundrum of immunoassays are harmonization and standardization of the assays for clinical applications [5]. Different manufactures of the commercial detection kits of tumor markers may design different binding sites/ epitopes for the immunoassays which leading to fluctuations on quality and challenge on the standardization. Moreover, variation in the induction of immune response in biological system also contributes to the uncertainty of the quality and the production suffers from lot-to-lot variation. In addition, a minimal perturbation of conformational epitopes on native proteins will cause a failure in probing against the corresponding antigen by a monoclonal antibody. Technically, the relatively low stability and short shelf life of immunoassays also post challenge for frequent calibrations and fluctuations of the results. All these limitations lead to an urgent need for the development of a standardized method to detect tumor markers, especially gastric cancer, for clinical applications.

Aptamers which are either DNA or RNA oligonucleotides, are capable of binding different targets with high affinity and selectivity. The method for screening specific aptamers is known as 'SELEX' (Systematic Evolution of Ligands by Exponential enrichment), which was first developed in 1990. This '*in vitro* selection' technique allows a simultaneous screening of individual nucleic acid molecules up to 10^15^ per selection for a particular target [6, 7]. Although a selection between aptamers and a target could be carried out and optimized under any condition, aptamers can be applied not only to diagnosis, but also to therapy[8, 9] such as Pegaptanib, which is an FDA approved anti-vascular endothelial growth factor (anti-VEGF) RNA aptamer for the treatment of an age-related macular degeneration [10]. The increasing interest in aptamers attributes to their versatility and superior properties. Aptamers are in general more stable than antibodies, and modification of aptamers can greatly enhance their functionality and extend their shelf life. The quality of aptamers in terms of reliability and reproducibility is more consistent because their synthesis and purification are robust under controllable settings in a machine. Furthermore, the selection and optimization of aptamers for large scale production are more simple and efficient than producing specific monoclonal antibodies. Thus, the selection for aptamers and their consequent application are rather promising in biological research and medical services.

Over the last decade, as the rapid progress of SELEX, developing aptamers for commercial and clinical interesting targets is becoming more and more efficient [11, 12]. SELEX has been successfully integrated in microfluidic chip based systems to screen aptamers against the protein targets with high affinities [11–18]. Moreover, Cell SELEX explores the expression of cell surface epitopes and distinguishes between different types of target cells [19–22]. High-throughput next-generation sequencing (NGS) and subsequent computational data analysis have also been integrated in SELEX to optimize aptamer discovery at a high resolution[11]. Sequencing reads are clustered and ranked in the order of frequencies. Candidate aptamers are further selected by a cut-off of read count [19, 23–27].

An increasing number of DNA/RNA aptamers targeting gastrointestinal cancer biomarkers and cancer cells have been discovered in recent years. RNA aptamer YJ-1 binds specifically to CEA-positive cells and inhibited homotypic aggregation, migration and invasion by CEA-positive cells in mice [28]. Moreover, two DNA aptamers bound to lgV-like N domain of CEA were found inhibiting cell adhesion properties of cancer cells [29]. DNA aptamer cy-apt20 was found targeting human gastric carcinoma AGS cells while showed minimal recognition to normal gastric epithelial GES-1 cells [22]. Eight colorectal cancer stem cells (CR-CSCs)/CRC-specific aptamers were reported and three of them showed high affinities towards their respective target cells [20]. However, aptamers targeting other gastrointestinal cancer biomarkers such as CA72-4 haven’t been reported yet.

In this study, we set out to screen the library of aptamer by SELEX for three biomarkers of gastrointestinal cancer, CEA, CA50 and CA72-4, respectively. We identified 6 novel RNA aptamers (two aptamers for each biomarker) using high-throughput sequencing technology and then measured their affinity by fluorescence spectroscopy. Their dissociation constants ranged from 16.5 to 156nM which implicates high affinities between the aptamers and the antigens. Intriguingly, the predicted secondary structures of RNA aptamers from each antigen showed significant structural similarity and immunostaining using CEA aptamers demonstrated positive fluorescent signal on gastric carcinomas AGS cells, suggesting the structural recognition between the aptamers and the antigens. Moreover, we found that the cell viability and growth rate of human colorectal cell line LS-174T were substantially decreased after transfected with the aptamers which may demonstrate a functional role in cancer development. These results suggest the potential clinical applications of these aptamers as a diagnostic or therapeutic tool for gastric cancer in the future since the nature of aptamers can overcome the limitations of immunoassays with better sensitivity and specificity.

## Materials and methods

### DNA oligos and antigens

**Fig 1:**
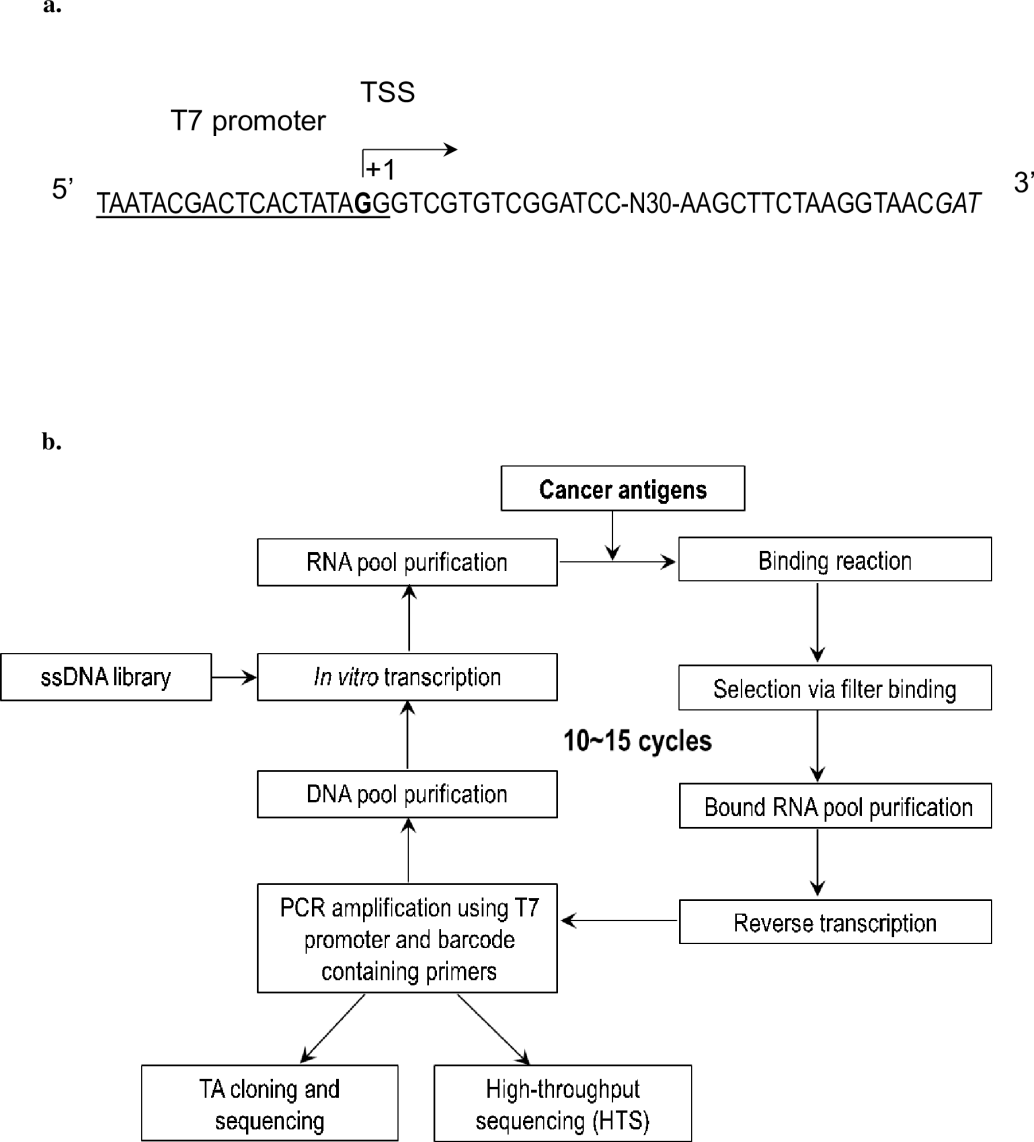
Designs of initial ssDNA library and SELEX workflow. **a)** Design and components of initial ssDNA library, underlined sequence at the 5’-end is the T7 promoter, the G in bold indicates the transcription start site (TSS), N30 refers random 30 nt in the viable region, the italic letters at the 3’-end are barcode adaptor for HTS; **b)** Workflow diagram of SELEX for screening aptamers, starting from a ssDNA library, ending at sequencing.

An initial DNA library that had a random 30-nucleotide sequence (N30) in between as the viable region, were deliberately designed and ordered (Life Technologies) as the template (Fig 1A). The length of N30 sequence is 82 nucleotides (nt), including a T7 promoter at the 5’-end, a *Bam*H I and a *Hin*d III restriction site flanking as the boundary, and a barcode sequence at the 3’-end for discrimination in sequencing. Gastrointestinal cancer biomarkers (Human source, high purity) were respectively purchased: CEA (Abcam), CA50 (BIO-RAD), and CA72-4 (BIO-RAD).

### SELEX and sequencing

SELEX, workflow shown in Fig 1B, was employed to screen RNA aptamers for all three gastrointestinal cancer biomarkers. The initial ssDNA library (5’-ATCGTTACCTTAGAAGCTT-N30-GGATCCGACACGACCCTATAGTGAGTCGTATTA-3’) was first transcribed into a starting RNA pool by pairing the T7 Initial-F primer (5’-AATACGACTCACTATAGGGTC-3’) and using *in vitro* transcription with T7 MEGAscript T7 Kit (Ambion) at 37°C for 6 hours. RNA product was subsequently purified by flowing through a Centri-Spin 20 column (PRINCETON). The starting RNA pool (~10^14^ sequences) was then incubated with a cancer antigen (~1 μg) in RSB-100 buffer (10 mM Tris-HCl, 5 mM MgCl_2_, 100 mM NaCl, 0.01% NP-40, pH 7.5) at room temperature for 30 min. Filter binding assay was performed to retain RNA-bound proteins on a PVDF membrane (GE Healthcare). Bound RNAs were released after the antigen was digested by proteinase K (Promega), and recovered through acid:phenol chloroform extraction and isopropanol precipitation. Recovered RNAs were subsequently amplified by RT-PCR using Superscript III First-Strand Synthesis System (Life Technologies) and iProof DNA polymerase (BIO-RAD) for 16–20 PCR cycles, followed by ethanol precipitation and *in vitro* transcription to transcribe dsDNA library to RNA library again to complete a SELEX cycle. Generally, the enrichment process was iteratively conducted for 10–15 cycles for selection of aptamers with elevated stringency through lowering antigen concentration, shortening incubation time, and increasing salt concentration of wash buffer. The final round of SELEX was stopped after RT-PCR. The corresponding purified DNA product was subsequently subjected to TA cloning with pGEM-T easy vector (Promega) and SANGER sequencing (BGI), as well as high-throughput sequencing (HTS) by ion Torrent sequencing platform (Life Technologies) when the RNA pool was enriched to a certain level. The quality and quantity of the same pool of DNA for ion Torrent was first verified by Agilent 2100 Bioanalyzer (Agilent Technologies). Such DNA pool was then applied to construct an amplicon library with the Ion Plus Fragment Library Kit (Ambion) according to manufacturer’s instructions, followed by sequencing on Ion 314 Chips (Life Technologies). Through a systematic bioinformatics analysis on the reads by Cygwin (Redhat), sequences were extracted and listed. Top ranking aptamers for each cancer biomarker were chosen for following biochemical characterization.

### Purification of RNA aptamers

To characterize top ranking aptamers, specific full length oligos, with replacement of N30 in the initial DNA library by identified sequences, were chemically synthesized for further study. Similarly, RNA aptamers were first generated through *in vitro* transcription using T7 MEGAscript Transcription Kit (Ambion), and purified on a 6% denaturing urea/polyacrylamide gel. The corresponding bands were visualized under UV, excised and chopped. RNA aptamers were eluted with 400 μl RNA elution buffer (0.5 M ammonium acetate, 1 mM EDTA, 0.1% SDS, pH 8.0) at 42°C overnight. Purified RNA aptamers were recovered through acid:phenol chloroform extraction, isopropanol precipitation, and dissolving in appropriate volume of RNase-free distilled water.

### Determination of binding profile

RNA aptamers were fluorescence labeled with Ulysis Alexa Fluor 488 nucleic acid labeling Kit (Life Technologies) following manufacturer’s instructions. Briefly, 1 μg ethanol precipitated gel purified RNA aptamers were mixed with 1 μl ULS reagent in DMSO and added up to 25 μl with labeling buffer. The mix was incubated at 90°C for 10 min, followed by stopping the reaction on ice. A Centri-Spin 20 column (PRINCETON) was applied to purify fluorescence labeled RNA. Next, samples for RNA-antigen interaction were prepared by incubating 1 pmol fluorescence labeled RNA and titrated with various concentrations of antigen (0, 10, 20, 50, 100, 200 nM) in RSB-100 buffer in the presence of non-specific competitor yeast tRNA (1ug/ul) at room temperature for 10 min respectively. Excitation fluorescence values at 516 nm were measured using Fluormax-4 Spectrofluorometer (Horiba), and fluorescence changes were obtained by subtracting the value in the absence of antigen [30]. Binding profiles were plotted and equilibrium dissociation constant K_*d*_ values were calculated using SigmaPlot v11.0 software (Systat). The error associated with the Kd values was calculated by the experiments in biological triplicate and each measurement was done in technical triplicate.

### Cell line and cell culture

The human colon adenocarcinoma cell line LS-174T, gastric adenocarcinoma cell line AGS and uterine cervical cancer cell line Hela used in this study were purchased from American type Culture Collection (ATCC). LS-174T cells were grown and maintained in culture flask with Dulbecco’s Modified Eagle Medium (DMEM) (Life Technologies) supplemented with 10% fetal bovine serum (FBS) and 1% penicillin/streptomycin (PS), incubating at 37°C in a 5% CO_2_ environment. For passage, cells were detached by trypsin, spun down, and resuspended in fresh DMEM with FBS and PS, followed by a 1/10 subculture in flask. AGS and Hela cells were grown and maintained in RPMI-1640 medium 10% FBS and 1% penicillin/streptomycin (PS), incubating at 37°C in a 5% CO_2_ environment.

### Transfection with aptamers

Cell density of LS-174T was determined by trypan blue (Life Technologies) staining. Around 4×10^5^ cells were seeded in a 6-well plate to reach 50% confluence overnight, and transfected with 1 μg gel purified selected RNA aptamer in 10 μl Lipofectamine 2000 (Life Technologies) following the manufacturer’s instructions. Antibiotic-free DMEM for transfection was replaced by 2 ml DMEM with FBS and PS after 5-hour incubation. The 48-hour post-transfected cells were detached by trypsin for following assays.

### Evaluation of cell viability and cell growth

Cell viability was determined by trypan blue exclusion. Briefly, 10 μl detached LS-174T cells were stained with 10 μl 0.4% trypan blue solution, and 10 μl mix was then loaded onto a hemocytometer. Numbers of viable cells (transparent) and dead cells (blue) were counted respectively under a light microscope at low magnification. Cell viability was calculated as the number of viable cells divided by the total number of cells within each 4×4 grid on the hemocytometer.

Cell growth rate was determined by MTT tetrazolium reduction assay. LS-174T cells treated with various RNA aptamers were seeded as 3000 cells/well in 96-well plates respectively. After 24, 48, 72 and 96-hour (as Day 1, 2, 3, 4) incubation, the plates were taken out one by one, and assayed with 10 μl MTT reagent mixture (5 mg/ml in PBS) incubating at 37°C for 4 hours. Absorbance at 570 nm was then measured by a Powerwave XS microplate reader (BioTek). Relative cell viability was calculated as A_570 nm on Day N_ divided by A_570 nm of Day 0_. The error was calculated by the experiments in biological triplicate and each measurement was done in technical triplicate.

### Immunostaining of gastric adenocarcinoma and uterine cervical cancer cell lines using RNA aptamer

AGS were grown on 10mm coverslip, harvested by washing with PBS for three times and fixed with cold methanol for 10 min. After washing three times with PBS, cells were incubated with binding buffer (BSA 1mg/ml in Dulbecco’s PBS without calcium and magnesium (pH 7.3), Mg_2_Cl 5mM, glucose 4.5g/L, Salmon Sperm DNA 0.1 mg/ml and yeast tRNA 0.1 mg/ml) for 30 min, followed by incubation of 1:100 RNA aptamer targeting CEA or 1:100 anti CEA, anti CA-50 and TAG72 antibodies (Abcam) in binding buffer for 1 hr. For secondarty antibody incubation, cells were washed with PBS for three times and incubated with 1:1000 rabbit anti-mouse/FITC antibody (Abcam) for 30 min. The cells were then washed three time with buffer, mounted, sealed and observed by fluorescence microscopy. All images were captured under a fluorescent microscopy with 400X magnification.

## Results

### RNA aptamers isolated for CEA, CA50 and CA72-4

A random library of N equal to 30 was used to screen RNA aptamers. The initial number of DNA template for *in vivo* transcription was set as approximately 10^14^, which means 10^14^ different RNA molecules in the starting RNA pool. This number of RNA copies which is equivalent to 2–5 μg covers the majority of the possible combinations of N30. In order to eliminate the non-specificity of the RNA to the PVDF membrane, a pre-clearing step was performed by immersing the membrane into the initially transcribed RNA pools for 15 min. The recovered RNA pool was then incubated with a cancer antigen to initiate SELEX.

Enrichment of RNA aptamers specific to cancer antigens was monitored by the binding ratio calculated before and after the binding experiment (amount of recovered RNA versus amount of input RNA). Through multiple rounds of SELEX, binding ratios of 18.1% for CEA at Round 14, 15.6% for CA50 at Round 12, and 24.6% for CA72-4 at Round 15 were recorded, indicating that specific RNA aptamers for cancer antigens were accumulated to a certain level, respectively.

In order to assess the sequences of enriched RNA aptamers after selection, the purified aptamers were reverse transcribed, amplified, subjected to overhang addition at 3' end and subcloned into the TA vector. For each antigen, 15–20 single colonies were randomly picked for sequencing. As shown in Table 1, the consensus sequences (*, **) were successfully identified in all three RNA pools, indicating the feasibility of the enrichment methodology and the possibility to subject for high-throughput sequencing.

**Table 1:** N30 RNA sequences identified by SANGER sequencing.

Sample	RNA Sequence	
CEA_R15-02	AGGCACGACGCAUAGCCUUGGGAGCGAGGA	*
CEA_R15-04	CCGCGAUCAUGACUACGCCCAUCCCCUUGG	
CEA_R15-05	AUGUAGGAUCGGCAACGUCCCGUACCUUGG	
CEA_R15-06	CCCGUCCACACACGGCCUGCCUGCCUUAGG	
CEA_R15-07	AGGCACGACGCAUAGCCUUGGGAGCGAGGA	*
CEA_R15-08	UUCUGCAGUUCUGGCACGACAUCCCCUUAG	**
CEA_R15-09	ACCGGCGCGUCACCCAACCUGGAGGCUACC	
CEA_R15-10	CGUGGGAGAUCUGGCCUACCCCCACCUUGG	
CEA_R15-11	CAACGGCACUGGCCCGCCCUUGGAGGCCUC	
CEA_R15-13	UCGAUCUGACGCGCGACCACCCGCCUUAGC	
CEA_R15-14	UUCUGCAGUUCUGGCACGACAUCCCCUUAG	**
CEA_R15-15	UCACCGGAUGCGGCGCUCCCCUAGCCUUGG	
CEA_R15-16	UACGGCAUGACCUAACCUGGAGGCGCAUCA	
CEA_R15-17	GCCUAGGAGCCAACCGUCCCCGCGCCUUGG	
CEA_R15-19	AGAGAUACGAUUCGGCCCGUGCCUUAGAGC	
CEA_R15-20	AGGCACGACGCAUAGCCUUGGGAGCGAGGA	*
CA50_R10-01	AGGGGGAAGUCCCCGGAACGGCGCAAUCCA	
CA50_R10-03	CUGGGGAACUGGCAGACCCAUUGCCUUAGA	*
CA50_R10-05	CGUGCCAGAGAGCAUGCGAAACGACAGACC	
CA50_R10-06	CUGGGGCACUGGCAGACCCAUUGCCUUAGA	
CA50_R10-07	CUGGGGAACUGGCAGACCCAUUGCCUUAGA	*
CA50_R10-08	CUGGGGAACUGGCAGACCCAUUGCCUUAGA	*
CA50_R10-09	CUGGGGAACUGGCAGACCCAUUGCCUUAGA	*
CA50_R10-10	AGCUCGAAAGUGGGCUGGCGAUGUGUCCCG	
CA50_R10-12	CUGGGGAACUGGCAGACCCAUUGCCUUAGA	*
CA72-4_R15-02	CCCAAAAAGGAUUGGGGCGUCUGCAUGACC	*
CA72-4_R15-06	UGCGAAGGGGGGCAGAGGUUUGACGCGAGA	**
CA72-4_R15-07	UGCGAAGGGGGGCAGAGGUUUGACGCGAGA	**
CA72-4_R15-08	CCCAAAAAGGAUUGGGGCGUCUGCAUGACC	*
CA72-4_R15-10	UGCGAAGGGGGGCAGAGGUUUGACGCGAGA	**
CA72-4_R15-11	UGCGAAGGGGGGCAGAGGUUUGACGCGAGA	**

*, ** Consensus sequences

### Highly abundant aptamers revealed by high-throughput sequencing (HTS)

**Fig 2:**
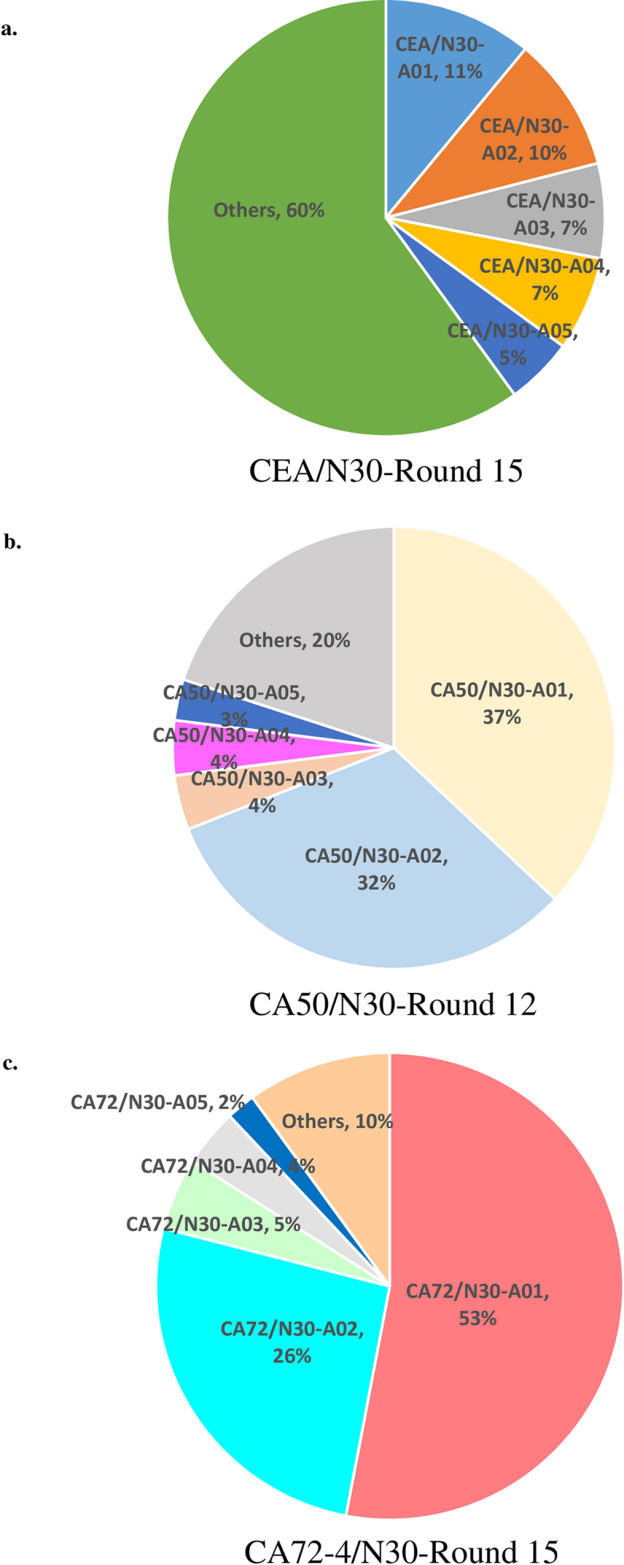
Distribution of top 5 N30 RNA sequences for CEA, CA50 and CA72-4 identified by HTS.

Since SANGER sequencing could only provide limited data, we adopted the high-throughput sequencing (HTS) to reveal the entire population of enriched RNA aptamers for the three antigens. To do this, RNA aptamers of CEA at Round 15, CA50 at Round 12, and CA72-4 at Round 15 were subjected to HTS using Ion Torrent system. The libraries were constructed and sequenced for each antigens according to the manufacturer protocols. The sequences were sorted and ranked based on their number of reads as shown in Table 2, the distribution of the identified aptamers was shown in Fig 2. In our results, the consensus sequences revealed by SANGER sequencing were all found in top 5 ranking sequences in HTS. In order to observe the profile of the aptamers in each case, the distribution of the aptamers was plotted as a pie chart. As shown in Fig 2 and Table 3, distributions of top 5 ranking sequences of CEA, CA50 and CA72-4 were up to 40%, 80% and 90% respectively, suggesting that these consensus sequences were enriched in SELEX experiments, and became dominant in the RNA pools because of their relatively high affinity to the antigens.

**Table 2:** Top 5 N30 RNA sequences identified by HTS.

Antigen	Ranking	Individual Reads / Effective Reads	RNA Sequence	
CEA	1	835 / 7633	AGGCACGACGCAUAGCCUUGGGAGCGAGGA	#
CEA	2	778 / 7633	AACGGCAUGACCUAACCUGGAGGCGCAUCA	
CEA	3	567 / 7633	AGAGAUACGAUUCGGCCCGUGCCUUAGAGC	
CEA	4	528 / 7633	CGGUAUCUGAUGCGGCCUGCUGCCUUAGAU	
CEA	5	361 / 7633	CAACGGCACUGGCCCGCCCUUGGAGGCCUC	
CA50	1	5613 / 14072	GUGAGUUUUUCGCGGCGAAGACAAGGCUCG	
CA50	2	4824 / 14072	AGCUCGAAAGUGGGCUGGCGAUGUGUCCCG	
CA50	3	576 / 14072	AGGGGGAAGUCCCCGGAACGGCGCAAUCCA	
CA50	4	549 / 14072	CUGGGGAACUGGCAGACCCAUUGCCUUAGA	#
CA50	5	530 / 14072	CCAGCGGAACGCACGAUCUGCCUUGGAAGC	
CA72-4	1	3995 / 7640	UGCGAAGGGGGGCAGAGGUUUGACGCGAGA	#
CA72-4	2	2000 / 7640	CCCAAAAAGGAUUGGGGCGUCUGCAUGACC	#
CA72-4	3	393 / 7640	ACAGACGUGGGCCGGGGCGGCCGAAAGAAA	
CA72-4	4	313 / 7640	CUCGGAGGCUUGCCUGCCGGGGGUGGCACG	
CA72-4	5	168 / 7640	UGCUGGAAAAACGCAGGUUUCGCUCGCCCG	

# Consensus sequences also identified by SANGER sequencing.

**Table 3:** Sequences and properties of selected RNA aptamers.

Aptamer	RNA Sequence (5’→3’)^*a*^	Length (nt)	MW (g/mol)^*b*^	ε (M^-1^·cm^-1^)^*b*^
CEA A01	GGGUCGUGUCGGAUCC**AGGCACGACGCAUAGCCUUGGGAGCGAGGA**AAGCUUCUAAGGUAACGAU	65	20051.8	623600
CEA A02	GGGUCGUGUCGGAUCC**AACGGCAUGACCUAACCUGGAGGCGCAUCA**AAGCUUCUAAGGUAACGAU	65	19916.7	615000
CA50 A01	GGGUCGUGUCGGAUCC**GUGAGUUUUUCGCGGCGAAGACAAGGCUCG**AAGCUUCUAAGGUAACGAU	65	19967.6	609300
CA50 A02	GGGUCGUGUCGGAUCC**AGCUCGAAAGUGGGCUGGCGAUGUGUCCCG**AAGCUUCUAAGGUAACGAU	65	19982.6	607000
CA72-4 A01	GGGUCGUGUCGGAUCC**UGCGAAGGGGGGCAGAGGUUUGACGCGAGA**AAGCUUCUAAGGUAACGAU	65	20148.8	626300
CA72-4 A02	GGGUCGUGUCGGAUCC**CCCAAAAAGGAUUGGGGCGUCUGCAUGACC**AAGCUUCUAAGGUAACGAU	65	19933.7	612500

^*a*^ Identified N30 sequence in the viable region was highlighted in bold letter

^*b*^ Calculated by IDT OligoAnalyzer 3.1 (1).

### Biochemical characterization of RNA aptamers

**Fig 3:**
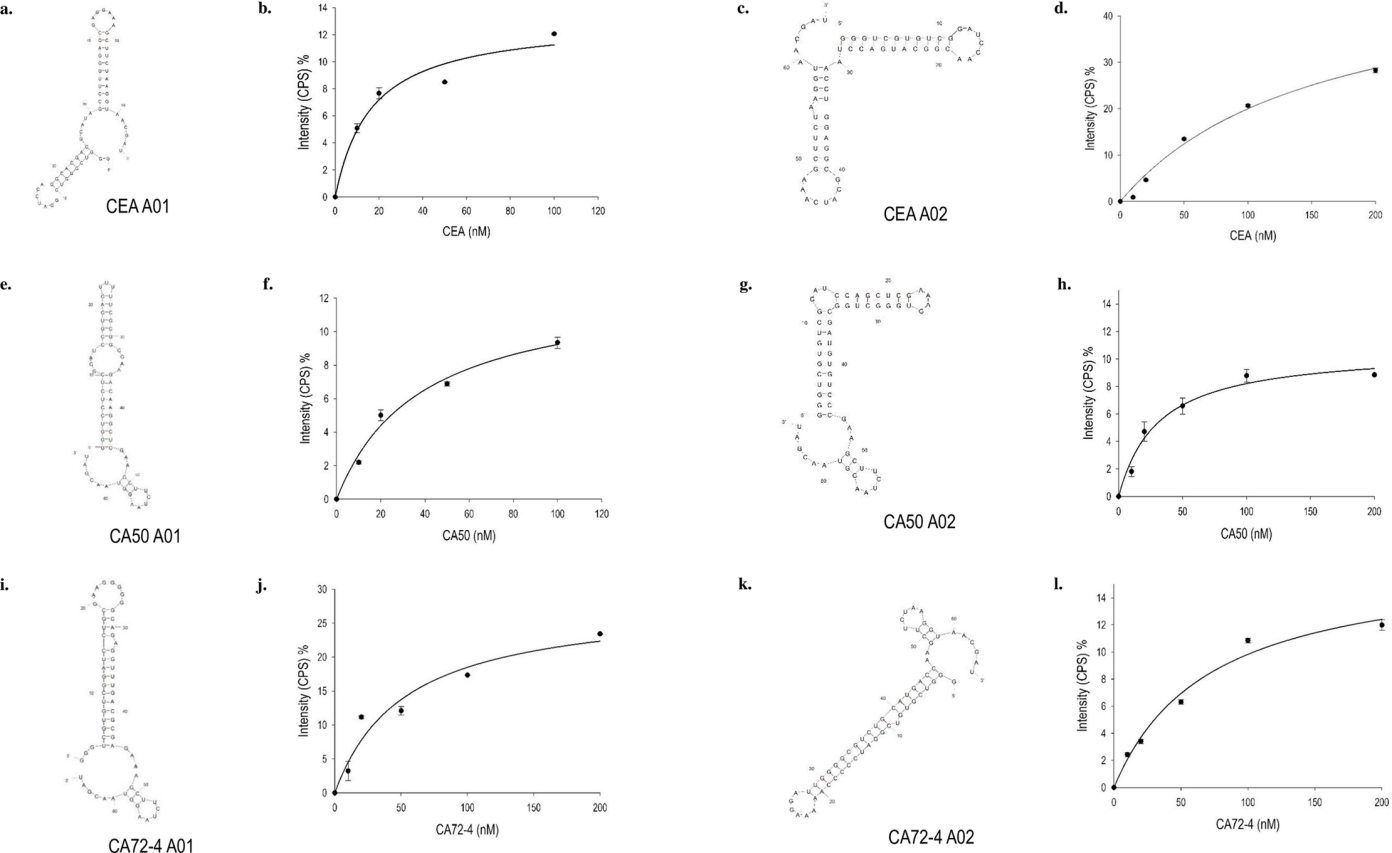
Secondary structure predicted by *mfold* (3), equilibrium dissociation curve and constant K_*d*_ of 6 RNA aptamers.

To characterize the RNA aptamers discovered by HTS, the top two RNA aptamers for each antigen were selected for further studies. The secondary structures of all six RNA aptamers, CEA A01, A02; CA50 A01, A02, and CA72 A01, A02 (Table 3), were predicted by *mfold* RNA Folding program [31]. As shown in Fig 3, interestingly, every two RNA aptamers for each biomarker exhibited similar folding and secondary structures without high sequence identity or similarity, suggesting that the aptamer structure may play an essential role in binding affinity and specificity. To evaluate these experimentally, we performed the titration assay using synthetic fluorescence labelled RNA aptamer to calculate their affinity to the corresponding antigens. The aptamers were *in vitro* transcribed, purified and labeled with fluorescence probe Alexa 488. The fluorescence labeled RNA aptamers were titrated with increasing amount of cancer antigens and the enhancement of fluorescence intensity was used to plot the binding isotherms. The curves were then fitted with the nonlinear regression—one site saturation ligand binding equation to obtain dissociation constant K_*d*_ as shown in Fig 3. Dissociation constant K_*d*_ values of CEA A01, A02 were 16.5 and 156 nM respectively; K_*d*_ values of CA50 A01, A02 was 38.0 and 30.7 nM respectively; K_*d*_ values for CA72 A01, A02 were 52.7 and 71.2 nM respectively (Table 4).

**Table 4:** Dissociation constants of the aptamer.

	Dissociation Constants, K_d_ (nM)	Regression of the fitting
CEA A01	16.5	0.98
CEA A02	156.0	0.99
CA50 A01	38.0	0.99
CA50 A02	30.7	0.99
CA72-4 A01	52.7	0.98
CA72-4 A02	71.2	0.99

1. Byrne DJ, Browning MC, Cuschieri A. CA72-4: a new tumour marker for gastric cancer. The British journal of surgery. 1990;77(9):1010–3.

2. Wang WS, Lin JK, Lin TC, Chiou TJ, Liu JH, Yen CC, et al. EIA versus RIA in detecting carcinoembryonic antigen level of patients with metastatic colorectal cancer. Hepato-gastroenterology. 2004;51(55):136–41.

3. Zuker M. Mfold web server for nucleic acid folding and hybridization prediction. Nucleic acids research. 2003;31(13):3406–15.

### Binding of CEA aptamers to gastric adenocarcinoma cell line AGS and uterine cervical cancer cell line Hela

**Fig 4:**
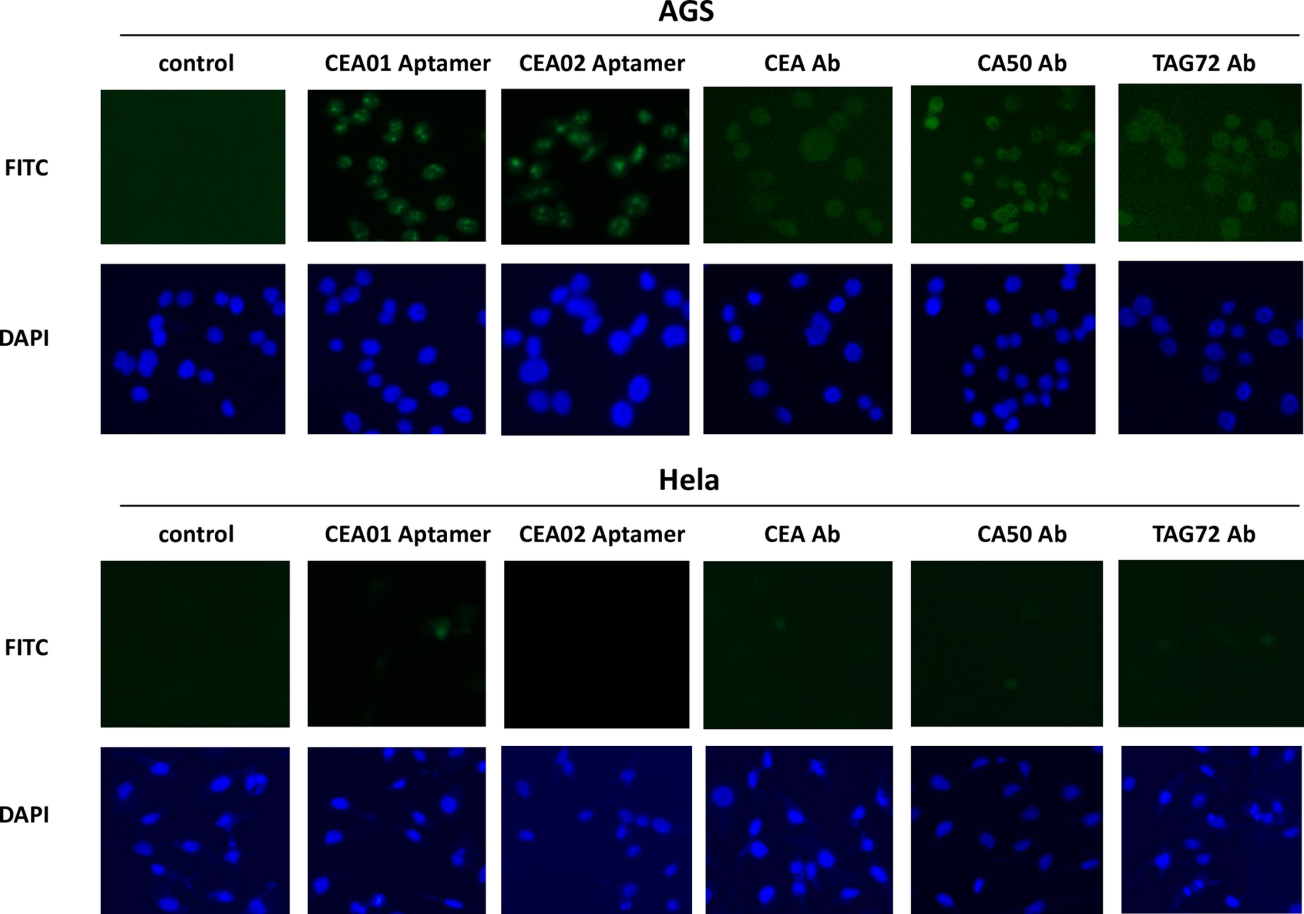
Immunostaining of AGS and Hela cells by using CEA aptamers probe (green), CEA, CA-50 and TAG72 antibodies (secondary with FITC) and DAPI (blue) stained the nucleus. All images were taken using a fluorescent microscopy with 400X magnification.

To investigate if the CEA aptamers can bind to cancer cells *in vitro*, immunostaining of AGS (antigen positive) and Hela (antigen negative/control) were performed. AGS cells incubated with CEA aptamers showed positive fluorescent signal similarly to CEA, CA-50 and TAG72 antibodies whereas Hela cells demonstrated no signal (Fig 4). The positive fluorescent signal of the immunostaining using aptamers comparable to the specific antibodies indicated the potential application of the aptamers as the cancer detection tool and the possibility to overcome the limitation of traditional immunoassays using unstandardized antibodies.

### Inhibitory effects of aptamers on LS-174T cells

**Fig 5:**
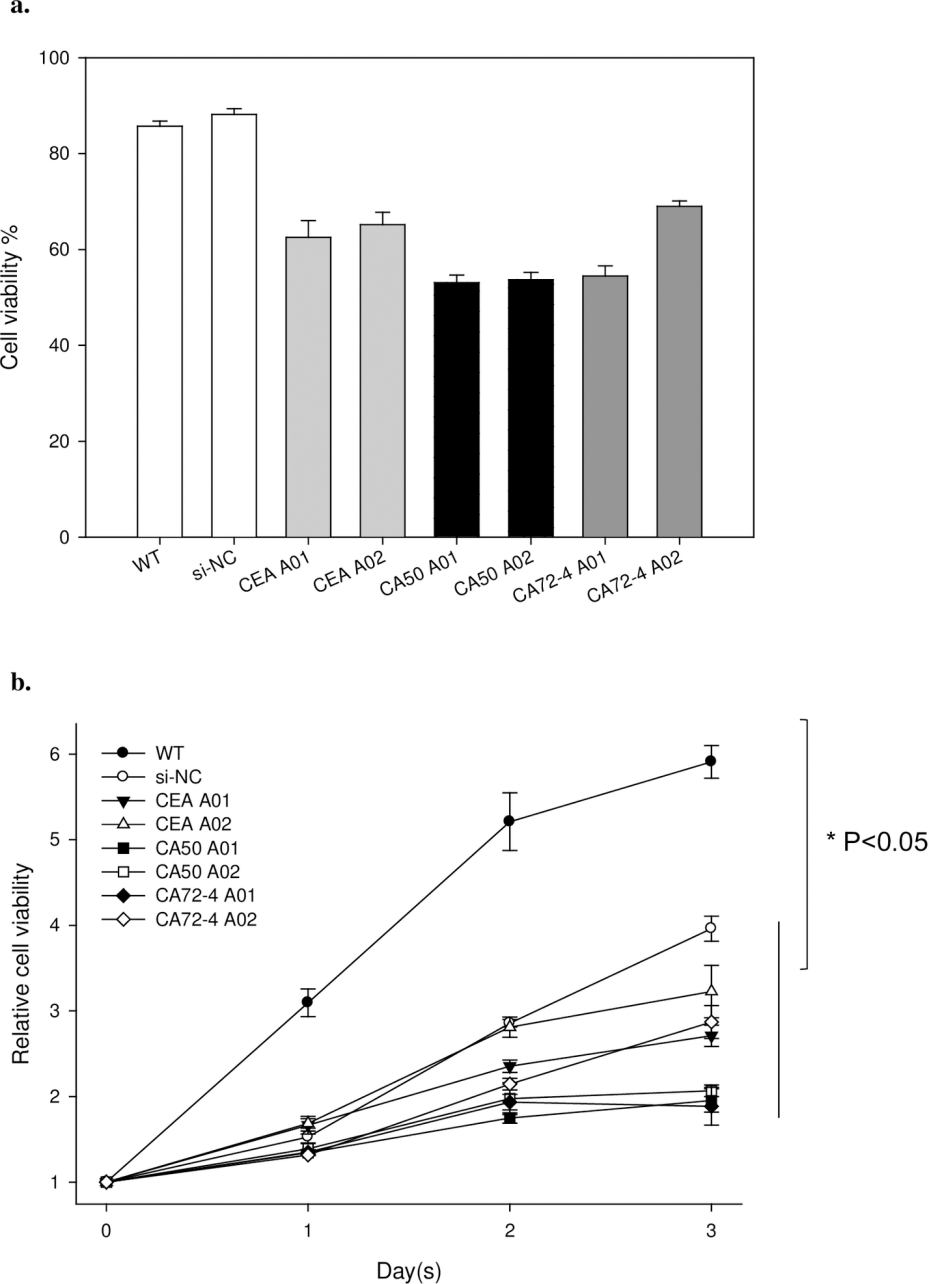
Inhibitory effects of selected aptamers on LS-174T cells. a) Cell viability determined by trypan blue staining 48-hour after the transfection; b) Relative cell growth rate monitored by the MTT assay from day 0 to day 3.

To explore the potential role of RNA aptamers, we transfected LS-174T cells with selected aptamers and studied cell viability and growth rate. The most valuable virtue of LS-174T cell line is that it produced all three cancer biomarkers CEA [32], CA50 [33] and CA72-4 [34] in cell, which facilitates our study towards the comparison of all three biomarkers. Cell viability was counted 48-hour after the transfection. Viable cells of both wild-type (WT, non-transfected) and negative control (si-NC, irrelevant siRNA) retained 85% and 87% (Fig 5A), indicating the transfection alone did not affect their growth and viability. On the other hand, viability of aptamer-treated cells greatly decreased to a range from 52% to 68% of total number of cell (Fig 5A). Moreover, study on the rate of cell growth demonstrated 2–3 folds suppression of aptamer-treated cells compared with WT after 3 days (Fig 5B), suggesting that the growth of LS174T cells was also inhibited by the aptamers.

## Discussion

To tackle the limitations of using immunoassays for the detection of tumor markers, we established the SELEX method incorporated with high-throughput sequencing by Ion Torrent system to screen RNA aptamers from a random RNA pool. Total 6 novel RNA aptamers for three biomarkers of gastrointestinal cancer, CEA, CA50 and CA72-4, were identified using SELEX and their biochemical properties were characterized. Although their affinities to each biomarker varied, they could reduce the viability and growth of tumor cells after transfection. Our pioneering development of SELEX to screen RNA aptamers against cancer biomarkers expanded the possibility of SELEX application as an alternative way to target such biomarkers besides antibodies.

One important improvement for our experiment is the application of high-throughput sequencing (HTS) for RNA SELEX. HTS techniques were developed and mature in recent years, which enabled researchers to screen efficiently and effectively, and profile nucleotide sequences within days. The compatibility of HTS in DNA SELEX was confirmed by several previous studies [14, 35], but it was not very commonly used in RNA SELEX. The application of HTS to inspect the RNA pool benefits us to globally view the whole population rather than an individual sequence, overcoming the shortage of insufficient information provided by limited SANGER sequencing [35]. In our study, HTS was carried out by Ion Torrent, which is a rapid, compact and economic technique for next generation sequencing. Top ranking sequences were obtained by subsequent statistical analysis, which clearly facilitate the selection process for promising aptamers. It is clear that this improvement undoubtedly opens a new pipeline to overview and designate aptamers for SELEX experiments.

The advantage of employing the human colorectal carcinoma cell line LS-174T as a model to study the potential role of RNA aptamers is that this cell line carries all three biomarkers of CEA [32], CA50 [33] and CA72-4 [34]. CEA are glycosyl phosphatidyl inositol (GPI) cell surface anchored glycoproteins, who may cooperate with CD44 variant isoforms to mediate colon carcinoma cell adhesion to E- and L-selectin [36]. CA50 and CA72 are both carbohydrate antigens with heavy glycosylations [4, 37], but their functions remained unclear. The diversity of glycosylation on cancer antigens varied their glycan compositions, molecular weights and structures [38], which might hamper the screening by SELEX for highly specific aptamers. Even so, HTS can facilitate the identification of highly abundant and most promising aptamers targeting the cancer biomarkers. The mechanism of the inhibitory effect on cell viability and growth by RNA aptamers is unclear and yet to be determined.

Furthermore, the potential applications of fluorescently-labelled aptamers are being widely studied and proven to be applicable on cancer therapy and diagnosis, such as intraoperative surgery and cancer-type specific in vivo non-invasive imaging [39]. In particular, a number of aptamers have been studied for their implications in specific recognition of cancer-related targets (e.g. thrombin, platelet-derived growth factor (PDGF), angiogenin, mucin, vascular endothelial growth factor (VEGF) and etc) [39]. In addition, the internalization process by which aptamers are uptake into cells depends on the function of their targets and typically divided into clathrin-mediated endocytosis or micropinocytosis [40]. The putative internalization mechanism of the selected aptamers of our study will be of great interest and clinical importance for further investigation as its mechanism of internalization in antigen positive cancer cells remains unknown. In this context, our recent study about the RNA aptamers targeting the cancer markers of CEA, CA50 and CA72.4, will give more impetus towards the development of the applications of aptamers as the therapeutic and/or diagnostic tools and the putative internalization mechanism may open the possibility for the aptamers as the targeted delivery agent for the discovery of specific anti-cancer drugs.

Taken together, this study demonstrates that SELEX incorporated with HTS is a promising and powerful tool to screen aptamers for various targets. To obtain qualified aptamers with high specificity against cancer antigens, SELEX refinement and post-SELEX development of aptamers are yet indispensable. In order to enhance the sensitivity and specificity of aptamers for future diagnostic and clinical applications, several modifications could be considered: to truncate ribonucleic acids in block to simplify the aptamer and specify the core region for RNA-protein interactions; to individually substitute / delete / insert a particular ribonucleic acid fragment in the core region of aptamer to improve its specificity; to chemically modify particular ribonucleic acid(s) of aptamer with locked nucleic acid (LNA) to decrease its susceptibility to nucleases and increase its stability for long-term storage. Indeed, it is possible that such aptamers would become applicable in clinical applications or even therapeutics in the near future with better sensitivity and specificity, and without the limitations of immunoassays.
